# Supplementary material for: High resolution and contrast 7 tesla MR brain imaging of the neonate
Source: Front Radiol. 2024 Jan 18;3:1327075. doi: 10.3389/fradi.2023.1327075 (PMC10830693; doi:10.3389/fradi.2023.1327075)
Supplement: Supplementary file 1 [file Table1.docx]

| **Sequence** | **Resolution (mm)** | **FOV Frequency** | **TR (ms)** | **TE (ms)** | **Acceleration factor** | **FA (deg)** | **Other Parameters** | **Acquisition Time (min)** | **Measured Sound Pressure Level (dBA)** |
| --- | --- | --- | --- | --- | --- | --- | --- | --- | --- |
| **Localizer** | 0.7x0.7x5.0 | 250 x 250mm | 4000 | 107 | GRAPPA 4 | 115 |  | 0:22 | 92.0 |
| **B0 map** | 5.0x5.0x5.0 | 210 x220mm | 10.0 | 1.02, 2.26, 4.08 | none | 10 |  | 019 | 95.0 |
| **T2 - Axial** | 0.6x0.6x1.2 | 141 x 151mm | 8640 | 156 | GRAPPA 2 | 120 | - | 2:37 | 93.3 |
| **T2 – Sagittal** | 0.6x0.6x1.2 | 154 x 125mm | 8640 | 156 | GRAPPA 2 | 120 | - | 3:12 | 90.8 |
| **T2 – Coronal** | 0.6x0.6x1.2 | 154 x 125mm | 8640 | 156 | GRAPPA 2 | 120 | - | 3:29 | 94.7 |
| **AFI** | 2.2x2.2x3.0 | 140 x 140 | 141 | 1.93, 3.62, 9.00 | GRAPPA 3 | 60 |  | 1:54 | 90.5 |
| **MRS (STEAM)** | 16x16x16 | - | 3000 | 20 | none | 90 | TM = 10ms | 2:45 | 60.0 |
| **BOLD fMRI (GRE-EPI)** | 1.0x1.0x1.0 | 125 x 125mm | 2560 | 43 | GRAPPA 3  Multiband 3 | 90 (exc)  110 (deg) | - | 7:26 | 94.2 |
| **Susceptibility Weighted Imaging (SWI)** | 0.2x0.2x1.2 | 122 x 150mm | 22 | 15 | GRAPPA 3 | 15 | - | 2:12 | 95.2 |

**Table 1: Acquisition sequence parameters.** (FOV: field of view; TR: repetition time; TE: echo time; FA: flip angle; TI: inversion time; GRAPPA: GeneRalized Autocalibrating Partial Parallel Acquisition; TM: mixing time; STEAM: STimulated Echo Acquisition Mode; AFI: Actual Flip-angle Imaging; GRE-EPI: Gradient echo Echo Planar Imaging)
